# Supplementary material for: Mapping and Candidate Gene Analysis of an All-Stage Stem Rust Resistance Gene in Durum Wheat Landrace PI 94701
Source: Plants (Basel). 2024 Aug 8;13(16):2197. doi: 10.3390/plants13162197 (PMC11359134; doi:10.3390/plants13162197)
Supplement: Supplementary file 1 [file plants-13-02197-s001.zip › plants-3112984-supplementary.pdf]

Supplementary Figures

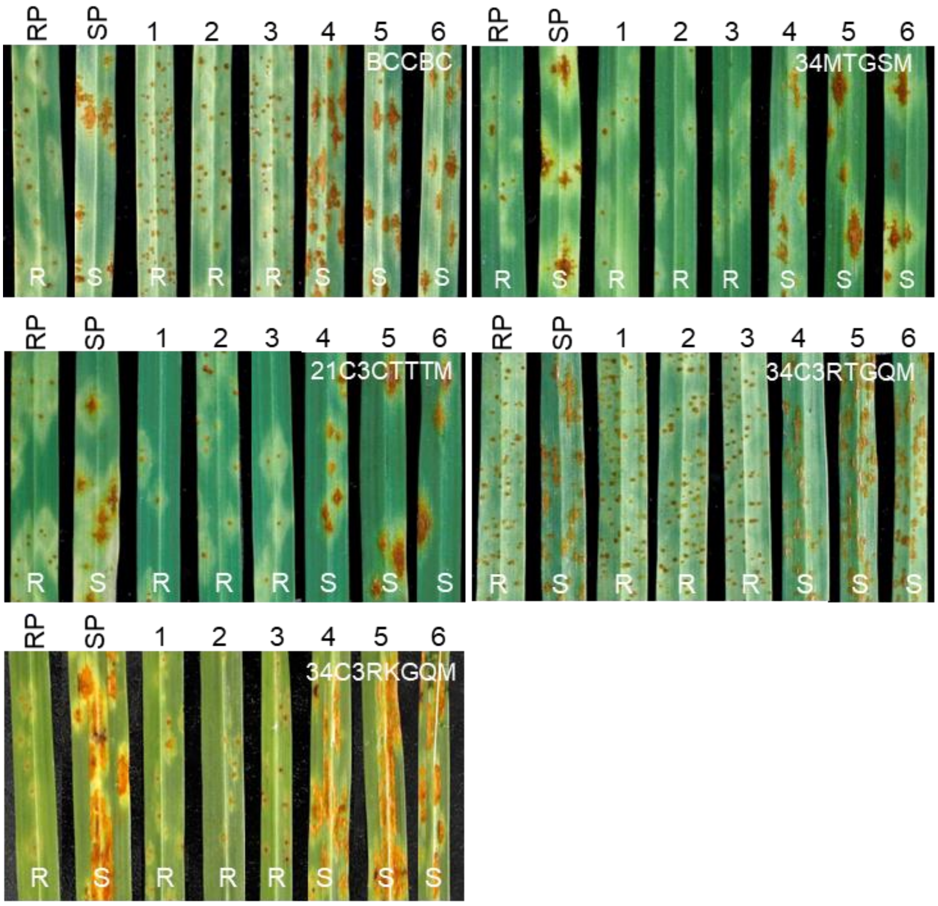

Figure S1 Infection types to *Pgt* races BCCBC, 34MTGSM, 21C3CTTTM, 34C3RTGQM, and 34C3RKGQM in $F_{2:3}$ families selected from the PI 94701 × Rusty cross. Plants were grown in five independent growth chambers at 22-24 °C with 16 h light/8 h dark. RP, resistant parent; SP, susceptible parent; 1-3, resistant F_3 plants; 4-6, susceptible F_3 plants; R, resistant; S, susceptible.

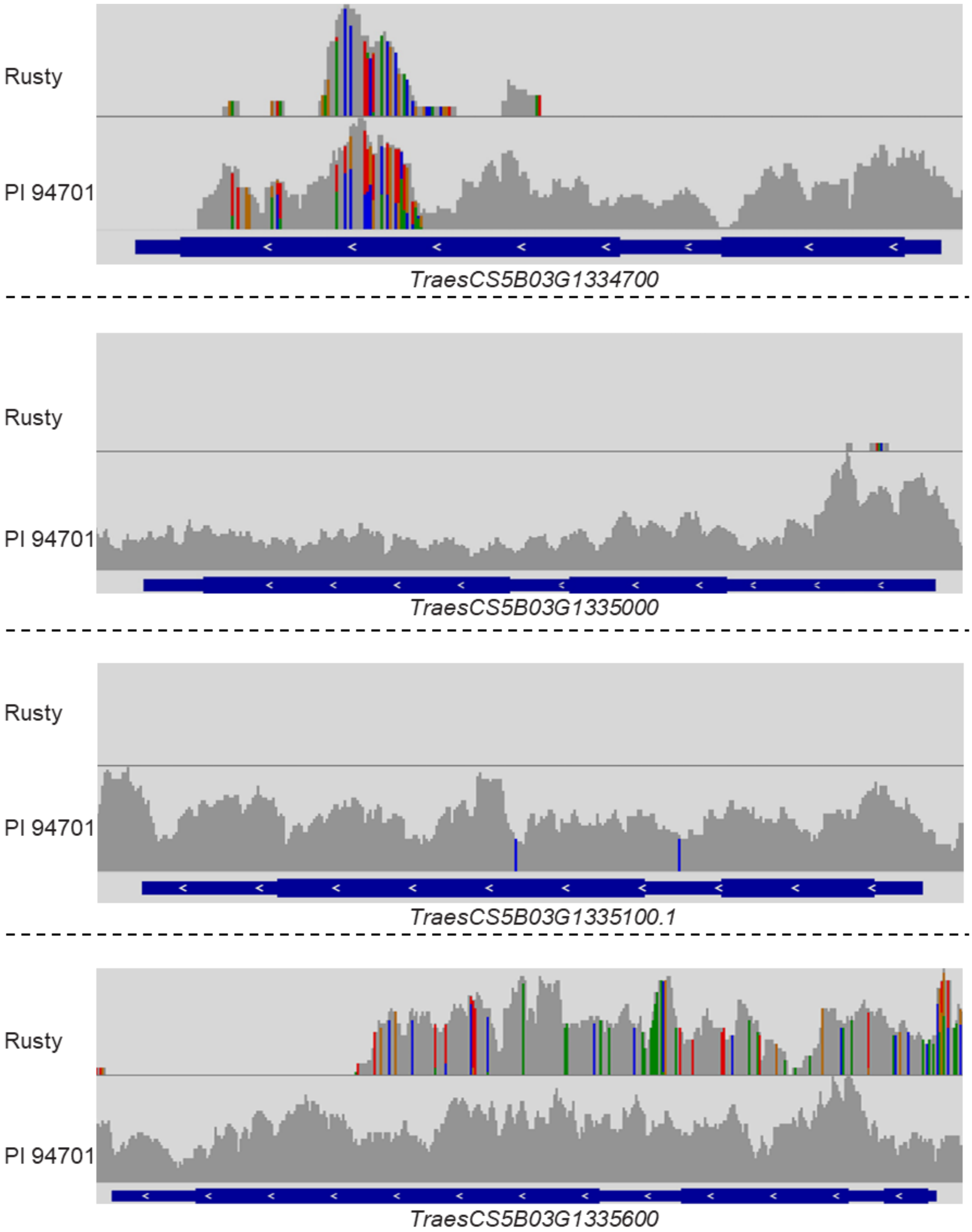

Figure S2 Comparison of the whole-genome resequencing reads from Rusty and PI 94701 for the candidate genes using the alignment algorithm BWA-MEM. For genes *TraesCS5B03G133500* and *TraesCS5B03G1335100*, no reads from Rusty were detected in the whole-genome sequencing data. The image illustrates sequence read numbers (Y-axis) and position in the candidate gene contigs (X-axis). Colored rectangles (blue) represent the gene structure.

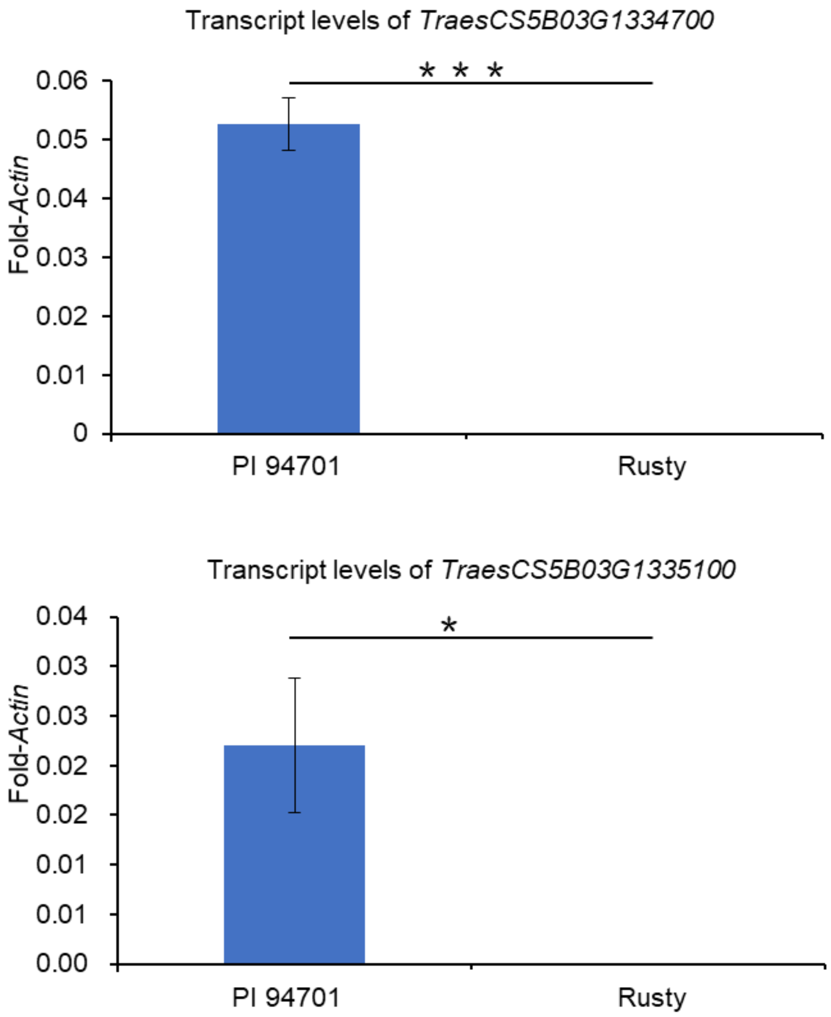

Figure S3 Transcript levels of the candidate genes *TraesCS5B03G1334700* and *TraesCS5B03G1335100* in F_4 sister lines S73 and R32. Seedlings of S73 and R32 were inoculated with *Pgt* race 34MKGQM and leaf samples from multiple plants were collected at 6 dpi. Transcript levels were expressed as fold-*Actin* (n = 4), and error bars represent standard errors of the mean.

Supplementary Tables

Table S1. Avirulence / virulence formulae of the *Puccinia graminis* f. sp. *tritici* races used in this study.

Race (isolate)	Origin	Avirulence	Virulence
BCCBC (09CA115-2)	USA	*SrPI94701 5 6 7b 8a 9a 9d 9e 9b 10 11 21 24 30 31 36 38 Tmp*	*Sr9g 17 McN*
34MTGSM (20GSA1)	China	*SrPI94701 8155b 9e 12 13 14 17 21 22 23 26 30 31 33 35 36 37 38 47 Tmp*	*Sr5 6 7b 8a 9a 9b 9d 9f 9g 10 11 15 16 18 19 20 24 25 27 28 29 32 34 39 McN*
34C3RTGQM (20IAL32)	China	*SrPI94701 8155b 9e 10 12 13 15 17 19 20 23 27 30 31 33 35 36 37 38 Tmp*	*Sr5 6 7b 8a 9a 9b 9d 9g 11 14 18 21 24 25 28 29 32 34 McN*
21C3CTTTM (20GH13)	China	*SrPI94701 8155b 5 9e 14 19 21 22 23 26 27 31 33 35 37 38 39 47*	*Sr6 7b 8a 9a 9b 9d 9f 9g 10 11 12 13 15 16 17 18 20 24 25 28 29 30 32 34 36 Tmp McN*
34MKGQM (20IAL06)	China	*SrPI94701 8155b 9e 10 11 13 14 17 18 19 21 22 23 25 26 30 31 33 34 35 36 37 38 47 Tmp*	*Sr5 6 7b 8a 9a 9b 9d 9f 9g 12 15 16 20 24 27 28 29 32 39 McN*
34C3RKGQM (21PK9)	China	*SrPI94701 8155b 10 11 13 14 15 17 18 20 22 25 30 31 33 34 35 36 37 Tmp*	*Sr5 8a 12 19 21 23 24 27 28 29 32 McN*

Table S2. SNPs linked with *SrPI94701* and their locations in the Svevo Rel.1.0.

Chromosome	Location (bp)	Allele	Rusty (SP)	PI 94701 (RP)	S-Bulk	R-Bulk
5B	673537026	A/C	A	C	A	C
5B	673538615	C/T	C	T	C	T
5B	673538642	A/G	A	G	A	G
5B	690132538	A/G	A	G	A	G
5B	690132553	T/C	T	C	T	C
5B	690132681	C/T	C	T	C	T
5B	690132702	T/C	T	C	T	C
5B	690132780	T/C	T	C	T	C
5B	690132918	A/G	A	G	A	G
5B	690133547	A/G	A	G	A	G
5B	690134308	T/C	T	C	T	C
5B	690134560	T/G	T	G	T	G
5B	690134566	A/C	A	C	A	C
5B	690134809	C/A	C	A	C	A
5B	690134913	G/A	G	A	G	A
5B	690450276	G/T	G	T	G	T
5B	690450564	T/C	T	C	T	C
5B	690450594	T/C	T	C	T	C
5B	690450645	C/G	C	G	C	G
5B	690451829	T/C	T	C	T	C
5B	690452397	G/A	G	A	G	A
5B	690452463	G/A	G	A	G	A
5B	690452487	C/T	C	T	C	T
5B	690452862	G/A	G	A	G	A
5B	690452906	T/C	T	C	T	C
5B	690452930	T/C	T	C	T	C
5B	690452960	C/T	C	T	C	T
5B	690548168	G/A	G	A	G	A
5B	690548198	C/G	C	G	C	G
5B	690548266	C/G	C	G	C	G
5B	690548320	G/C	G	C	G	C
5B	690548365	G/A	G	A	G	A
5B	690549265	A/G	A	G	A	G
5B	690549283	T/C	T	C	T	C
5B	690550559	C/T	C	T	C	T
5B	690696603	T/C	T	C	T	C
5B	690696608	G/A	G	A	G	A
5B	690696623	G/C	G	C	G	C
5B	690696749	C/T	C	T	C	T
5B	690696846	T/C	T	C	T	C
5B	690699742	C/G	C	G	C	G

5B	690700002	A/G	A	G	A	G
5B	690700038	A/G	A	G	A	G
5B	690700079	T/A	T	A	T	A
5B	690700693	G/A	G	A	G	A
5B	691140854	T/C	T	C	T	C
5B	691140864	G/A	G	A	G	A
5B	691140907	T/C	T	C	T	C
5B	691140978	A/G	A	G	A	G
5B	691142510	A/G	A	G	A	G
5B	691142522	G/A	G	A	G	A
5B	691142606	C/G	C	G	C	G
5B	691142630	G/C	G	C	G	C
5B	691142744	C/G	C	G	C	G
5B	691142884	G/C	G	C	G	C
5B	691142990	C/T	C	T	C	T
5B	691153376	G/A	G	A	G	A
5B	691155372	C/T	C	T	C	T
5B	691158423	A/G	A	G	A	G
5B	691162721	C/T	C	T	C	T
5B	691163268	T/C	T	C	T	C
5B	691453615	T/C	T	C	T	C
5B	691454084	C/T	C	T	C	T
5B	691455997	A/G	A	G	A	G
5B	691456015	C/T	C	T	C	T
5B	691456471	A/G	A	G	A	G
5B	691456611	A/G	A	G	A	G
5B	691456672	T/C	T	C	T	C
5B	691456696	G/T	G	T	G	T
5B	691456859	T/C	T	C	T	C
5B	691460240	G/A	G	A	G	A
5B	691460596	G/A	G	A	G	A
5B	691460602	A/T	A	T	A	T
5B	691460806	A/G	A	G	A	G
5B	691460858	C/A	C	A	C	A
5B	691676385	A/G	A	G	A	G
5B	691676406	A/G	A	G	A	G
5B	691676445	G/A	G	A	G	A
5B	691676493	C/G	C	G	C	G
5B	691676500	T/C	T	C	T	C
5B	691679615	G/C	G	C	G	C
5B	691679621	G/C	G	C	G	C
5B	691685590	G/A	G	A	G	A
5B	691685656	G/A	G	A	G	A
5B	691870564	C/T	C	T	C	T

5B	691870707	G/A	G	A	G	A
5B	691874455	G/A	G	A	G	A
5B	691874927	T/C	T	C	T	C
5B	691875084	A/C	A	C	A	C
5B	691875126	T/C	T	C	T	C
5B	691875285	C/T	C	T	C	T
5B	695900839	G/A	G	A	G	A
5B	695900896	C/T	C	T	C	T
5B	695901007	T/C	T	C	T	C
5B	695905265	C/T	C	T	C	T
5B	695905408	C/G	C	G	C	G
5B	695905497	C/A	C	A	C	A
5B	695905514	A/C	A	C	A	C
5B	695905562	C/T	C	T	C	T
5B	695905802	G/C	G	C	G	C
5B	695905892	A/G	A	G	A	G
5B	695905974	A/G	A	G	A	G
5B	696004071	A/G	A	G	A	G
5B	696004590	C/G	C	G	C	G
5B	696004614	G/A	G	A	G	A
5B	696006223	C/T	C	T	C	T
5B	696012374	G/C	G	C	G	C
5B	696012468	G/A	G	A	G	A
5B	696012965	C/A	C	A	C	A
5B	696013709	T/G	T	G	T	G
5B	696024115	A/G	A	G	A	G
5B	696027510	C/G	C	G	C	G
5B	696029048	T/C	T	C	T	C
5B	696375467	G/A	G	A	G	A
5B	696558983	T/C	T	C	T	C
5B	697067391	G/A	G	A	G	A
5B	697067436	A/C	A	C	A	C
5B	697093006	G/C	G	C	G	C
5B	697098140	G/A	G	A	G	A
5B	697412714	C/T	C	T	C	T
5B	697413013	C/T	C	T	C	T
5B	697413096	G/C	G	C	G	C
5B	697413597	T/A	T	A	T	A
5B	697413616	G/A	G	A	G	A
5B	697415781	G/T	G	T	G	T
5B	697415795	C/G	C	G	C	G
5B	697415801	T/C	T	C	T	C
5B	697416098	G/A	G	A	G	A
5B	697416206	G/A	G	A	G	A

5B	697416227	T/C	T	C	T	C
5B	697416254	A/T	A	T	A	T
5B	697416305	A/G	A	G	A	G
5B	697416402	G/A	G	A	G	A
5B	697416416	G/A	G	A	G	A
5B	698037709	A/G	A	G	A	G
5B	698039600	A/G	A	G	A	G
5B	698194607	A/G	A	G	A	G
5B	698194686	T/C	T	C	T	C
5B	698195129	T/C	T	C	T	C
5B	698195167	G/A	G	A	G	A
5B	698195375	T/G	T	G	T	G
5B	698809491	C/G	C	G	C	G
5B	698809555	A/G	A	G	A	G
5B	698827028	T/C	T	C	T	C
5B	698828178	G/A	G	A	G	A
5B	698832399	C/T	C	T	C	T
5B	698834478	G/C	G	C	G	C
5B	698834524	G/A	G	A	G	A
5B	698834615	T/C	T	C	T	C
5B	698834843	G/C	G	C	G	C
5B	699873281	T/C	T	C	T	C
5B	699873308	C/T	C	T	C	T
5B	699873320	G/A	G	A	G	A
5B	699873359	G/A	G	A	G	A
5B	699873389	G/T	G	T	G	T
5B	699874865	C/G	C	G	C	G
5B	699874977	C/T	C	T	C	T
5B	699936236	C/A	C	A	C	A
5B	699936256	A/C	A	C	A	C
5B	699936373	C/G	C	G	C	G

Table S3. Predicted genes within the *SrPI94701* candidate region based on the genomic sequence of Svevo Rel.1.0. NBS-LRR (NLR) genes are highlighted in blue and protein kinases are highlighted in green.

Gene ID in Svevo Rel.1.0	Location(bp)	Gene annotation
TRITD5Bv1G248710	691246432:691253036	NBS-LRR Disease resistance protein
TRITD5Bv1G248720	691256419:691257925	Protein FAR1-RELATED SEQUENCE 5
TRITD5Bv1G248730	691257962:691258753	Protein FAR1-RELATED SEQUENCE 5
TRITD5Bv1G248740	691282213..691282788	Pol polyprotein
TRITD5Bv1G248750	691306412:691306741	Gag-pol polyprotein
TRITD5Bv1G248760	691338839:691341573	Acyl-[acyl-carrier-protein] desaturase
TRITD5Bv1G248770	691339761:691340111	—
TRITD5Bv1G248780	691370776:691371072	Retrotransposon protein
TRITD5Bv1G248790	691436922:691437227	Transposon protein
TRITD5Bv1G248800	691454049:691460239	Transducin/WD-like repeat-protein
TRITD5Bv1G248810	691501161:691501856	Retrovirus-related Pol polyprotein from transposon TNT 1-94 TE
TRITD5Bv1G248820	691503752:691504072	Potassium voltage-gated channel subfamily V member 1 G
TRITD5Bv1G248830	691514447:691514833	Transposon protein
PTRITDBv1G248840	691538292:691538983	Acyl-[acyl-carrier-protein] desaturase
TRITD5Bv1G248850	691539040:691539351	Acyl-[acyl-carrier-protein] desaturase
TRITD5Bv1G248860	691548302:691548911	Hydroxyproline-rich glycoprotein family protein G
TRITD5Bv1G248870	691559538:691560569	Ethylene-responsive transcription factor
TRITD5Bv1G248880	691566157:691566906	E3 ubiquitin-protein ligase pub1 G
TRITD5Bv1G248890	691595319:691595885	Ethylene-responsive transcription factor
TRITD5Bv1G248900	691599040:691599384	Retrovirus-related Pol polyprotein from transposon TNT 1-94 TE
TRITD5Bv1G248910	691599928:691600257	Reverse transcriptase
TRITD5Bv1G248920	691600525:691600935	Retrovirus-related Pol polyprotein from transposon TNT 1-94 TE
TRITD5Bv1G248930	691601784:691602509	Retrovirus-related Pol polyprotein from transposon TNT 1-94
TRITD5Bv1G248940	691657895:691658254	RNA-dependent RNA polymerase 6 G
TRITD5Bv1G248950	691659748:691660071	NAD(P)-binding Rossmann-fold superfamily protein G
TRITD5Bv1G248960	691666704:691668634	Hydroxyproline-rich glycoprotein family protein G
TRITD5Bv1G248970	691670573:691671654	Pentatricopeptide repeat-containing protein
TRITD5Bv1G248980	691672790:691673122	Polynucleotidyl transferase
TRITD5Bv1G248990	691676349:691680291	Derlin; Ahrd_quality_code
TRITD5Bv1G249000	691685567:691689823	DNA polymerase epsilon subuni
TRITD5Bv1G249010	691690959:691691510	Myb domain protein
TRITD5Bv1G249020	691732804:691733645	Retrotransposon protein
TRITD5Bv1G249030	691735599:691736756	Retrovirus-related Pol polyprotein from transposon TNT 1-94
TRITD5Bv1G249040	691815441:691815758	Retrotransposon protein
TRITD5Bv1G249050	691839589:691840263	Hydroxyproline-rich glycoprotein G
TRITD5Bv1G249060	691863162:691866572	Receptor-like protein kinase
TRITD5Bv1G249070	691868660:691868971	Gamma-glutamyltranspeptidase 3 G
TRITD5Bv1G249080	691870608:691872047	Alanine--tRNA ligase G
TRITD5Bv1G249090	691872523:691874891	Ycf20-like protein

TRITD5Bv1G249100	691883573:691883971	Cytoplasmic FMR1-interacting protein 1 G
TRITD5Bv1G249110	691890931:691891428	GRF zinc finger family protein TE?
TRITD5Bv1G249120	691894360:691894905	Transposon protein
TRITD5Bv1G249130	691902419:691902742	Retrovirus-related Pol polyprotein from transposon TNT 1-94
TRITD5Bv1G249140	691930058:691930432	Retrovirus-related Pol polyprotein from transposon TNT 1-94
TRITD5Bv1G249150	691943897:691944693	Homeodomain-like superfamily protein G
TRITD5Bv1G249160	691945345:691945959	Ulp1 protease family
TRITD5Bv1G249170	691946772:691950677	tRNA-dihydrouridine(20/20a) synthase G
TRITD5Bv1G249180	691950741:691951342	Lipoxygenase G
TRITD5Bv1G249190	691958543:691958899	Werner Syndrome-like exonuclease
TRITD5Bv1G249200	691973655:691974086	Retrotransposon protein
TRITD5Bv1G249210	692004485:692019358	Flavin-containing monooxygenase
TRITD5Bv1G249230	692056751:692057218	—
TRITD5Bv1G249240	692080884:692081324	Transcription elongation factor SPT5 G
TRITD5Bv1G249250	692095406:692096778	Retrovirus-related Pol polyprotein from transposon TNT 1-94
TRITD5Bv1G249260	692105390:692107005	Ankyrin repeat family protein
TRITD5Bv1G249270	692107641:692108152	Ankyrin repeat family protein
TRITD5Bv1G249280	692110261:692117686	Ankyrin repeat family protein
TRITD5Bv1G249290	692164235:692164801	Retrotransposon protein
TRITD5Bv1G249300	692179316:692179690	Retrovirus-related Pol polyprotein from transposon TNT 1-94
TRITD5Bv1G249310	692180146:692180550	Retrovirus-related Pol polyprotein from transposon TNT 1-94
TRITD5Bv1G249320	692180838:692181200	Retrovirus-related Pol polyprotein from transposon TNT 1-94
TRITD5Bv1G249330	692190354:692191598	Myosin-10 G; ID=TRITD5Bv1G249330.1
TRITD5Bv1G249340	692197169:692197468	3-hydroxy-3-methylglutaryl-coenzyme A reductase G
TRITD5Bv1G249350	692257785:692271857	Methyl-CpG-binding domain-containing protein
TRITD5Bv1G249360	692280840:692283572	F-box family protein

Table S4. Predicted genes in the *SrPI94701* candidate region based on the genomic sequence of Chinese Spring (RefSeq v2.1). NBS-LRR (NLR) genes are highlighted in blue and protein kinases are highlighted in green. The expression levels of the candidate genes were based on published RNAseq studies compiled in the wheat expVIP database (http://www.wheat-expression.com/).

**Gene ID in CS RefSeq v2.1**	**Start**	**END**	**Strand**	**Function description**	**Expression or not**
TraesCS5B03G1334300	702.560373Mb	702.564756Mb	-	Disease resistance protein RPM1	Not
TraesCS5B03G1334500	702.61405Mb	702.616221Mb	+	Leucine-rich repeat receptor kinase-like protein	Not
TraesCS5B03G1334600	702.618368Mb	702.618688Mb	+	Protein kinase family protein	Not
TraesCS5B03G1334700	702.631769Mb	702.635441Mb	-	Disease resistance protein RPM1	Yes
TraesCS5B03G1334900	702.643865Mb	702.645383Mb	+	Receptor-like protein kinase	Not
TraesCS5B03G1335000	702.677875Mb	702.68294Mb	-	Disease resistance protein	Yes
TraesCS5B03G1335100	702.716212Mb	702.720447Mb	-	Disease resistance protein RPM1	Yes
TraesCS5B03G1335200	702.754213Mb	702.755609Mb	+	Werner Syndrome-like exonuclease	Not
TraesCS5B03G1335300	702.785489Mb	702.790804Mb	-	Disease resistance protein RPM1	Yes
TraesCS5B03G1335500	702.844686Mb	702.848323Mb	+	Receptor-like protein kinase	Yes
TraesCS5B03G1335600	702.852439Mb	702.8582Mb	-	Disease resistance protein (NBS-LRR class) family	Yes
TraesCS5B03G1335800	702.911476Mb	702.915825Mb	-	Disease resistance protein RPM1	Not
TraesCS5B03G1335900	703.642969Mb	703.644782Mb	-	Acyl-[acyl-carrier-protein] desaturase	Not
TraesCS5B03G1336100	703.808523Mb	703.81611Mb	+	Transducin/WD-like repeat-protein	Yes
TraesCS5B03G1336800	704.098827Mb	704.099421Mb	-	Hydroxyproline-rich glycoprotein family protein	Not
TraesCS5B03G1337300	704.194205Mb	704.194931Mb	+	Ethylene-responsive transcription factor	Not
TraesCS5B03G1337400	704.219804Mb	704.220353Mb	-	Hydroxyproline-rich glycoprotein family protein	Yes
TraesCS5B03G1337600	704.223674Mb	704.225128Mb	-	Pentatricopeptide repeat-containing protein, putative	Yes
TraesCS5B03G1337700	704.226305Mb	704.230813Mb	-	Derlin	Yes
TraesCS5B03G1337800	704.236306Mb	704.241341Mb	+	Nuclear factor Y, subunit C10	Yes
TraesCS5B03G1338000	704.242878Mb	704.245073Mb	+	Protein kinase family protein	Yes
TraesCS5B03G1338200	704.361881Mb	704.362863Mb	-	Hydroxyproline-rich glycoprotein	Yes
TraesCS5B03G1338300	704.387634Mb	704.391029Mb	+	Receptor-like protein kinase	Yes
TraesCS5B03G1338400	704.402336Mb	704.404802Mb	+	Ycf20-like protein	Yes
TraesCS5B03G1338800	704.414913Mb	704.416601Mb	+	Flavin-containing monooxygenase	Not
TraesCS5B03G1339700	704.517287Mb	704.525047Mb	-	Ankyrin repeat family protein	Yes
TraesCS5B03G1340100	704.695036Mb	704.699363Mb	+	Methyl-CpG-binding domain-containing protein 9	Not
TraesCS5B03G1340200	704.708346Mb	704.711203Mb	-	F-box family protein	Not

Table S5. Differentially expressed genes (DEGs) in the *SrPI94701* candidate region. TPM (transcripts per million) is used to measure expression levels. Differentially expressed genes (FDR < 0.05; *p*-value < 0.05; and |log2foldchange| > 1) are highlighted in bold.

**Gene ID**	**Resistant groups**			**Susceptible groups**			**|log2FoldChange|**	***p*-value**	**FDR**	**Annotation**
	**R32-1**	**R32-2**	**R32-3**	**S73-1**	**S73-2**	**S73-3**				
TraesCS5B03G1334700	**2.20**	**2.10**	**2.42**	**0.00**	**0.00**	**0.00**	**10.28**	**0.00**	**0.00**	**Disease resistance protein RPM1**
TraesCS5B03G1335000	**2.47**	**1.95**	**1.54**	**0.00**	**0.00**	**0.00**	**10.16**	**0.00**	**0.00**	**Disease resistance protein**
TraesCS5B03G1335100	**0.24**	**0.24**	**0.19**	**0.00**	**0.00**	**0.00**	**6.95**	**0.00**	**0.00**	**Disease resistance protein RPM1**
TraesCS5B03G1335500	3.94	0.08	2.81	2.59	1.85	1.92	0.26	0.83	0.96	Receptor-like protein kinase
TraesCS5B03G1335600	**1.24**	**1.49**	**0.95**	**0.30**	**0.44**	**0.33**	**1.55**	**0.00**	**0.00**	**Disease resistance protein (NBS-LRR class) family**
TraesCS5B03G1336100	**10.32**	**11.01**	**8.68**	**2.97**	**2.85**	**3.99**	**1.34**	**0.00**	**0.00**	**Transducin/WD-like repeat-protein**
TraesCS5B03G1337400	1.04	2.82	2.37	0.00	1.14	0.25	2.09	0.06	0.33	Hydroxyproline-rich glycoprotein family protein
TraesCS5B03G1337600	**0.40**	**0.77**	**0.41**	**0.00**	**0.08**	**0.02**	**3.82**	**0.00**	**0.01**	**Pentatricopeptide repeat-containing protein**
TraesCS5B03G1337700	**42.32**	**42.22**	**43.80**	**9.60**	**10.22**	**11.34**	**1.77**	**0.00**	**0.00**	**Derlin**
TraesCS5B03G1337800	1.61	1.79	1.22	1.29	1.17	1.43	0.03	0.93	0.99	Nuclear factor Y, subunit C10
TraesCS5B03G1338000	0.47	0.18	0.18	0.08	0.13	0.00	1.67	0.16	0.54	Protein kinase family protein
TraesCS5B03G1338200	1.94	6.57	6.13	2.03	2.76	1.66	0.93	0.08	0.39	Hydroxyproline-rich glycoprotein
TraesCS5B03G1338400	6.65	7.25	8.52	7.97	7.07	7.60	0.30	0.36	0.76	Ycf20-like protein
TraesCS5B03G1339700	0.83	0.47	0.06	0.13	0.54	0.18	0.48	0.60	0.89	Ankyrin repeat family protein
TraesCS5B03G1340200	**0.08**	**0.00**	**0.06**	**0.55**	**0.96**	**0.48**	**4.10**	**0.00**	**0.00**	**F-box family protein**

Table S6. Genotyping results for markers *pku69124*, *pku69187*, *pku69211*, *pku69227*, and *pku69228*. A collection comprising 53 *T. dicoccon* accessions and 51 *T. aestivum* genotypes was used in this study. Wheat accessions carry the *SrPI94701* haplotype are highlighted in green. NT, not test.

Accessions	Species	Markers					*Pgt* race
		pku69124 **(*Ssp*I)**	***pku69187*** **(*Bbv*CI)**	***pku69211*** **(*HpyCH4*IV)**	***pku69227*** **(*Pvu* II)**	***pku69228*** **(*Hpy188*III)**	**34MKGQM**
**PI 94701**	***T. durum***	**A**	**A**	**A**	**A**	**A**	1+/2-
**Rusty**	***T. durum***	**B**	**B**	**B**	**B**	**B**	3+
CItr 12214	*T. dicoccon*	A	A	A	A	A	1+
CItr 14621	*T. dicoccon*	A	A	H	A	A	2/2+
CItr 14637	*T. dicoccon*	A	A	A	A	A	1+
CItr 14919	*T. dicoccon*	A	A	H	A	A	1-
CItr 14970	*T. dicoccon*	A	A	A	A	A	1+
PI 94615	*T. dicoccon*	A	B	B	A	A	0;/1-
PI 94616	*T. dicoccon*	A	B	A	A	A	0
PI 94626	*T. dicoccon*	A	B	A	A	A	0;
PI 94631	*T. dicoccon*	A	A	A	A	A	1+/2
PI 94638	*T. dicoccon*	A	B	A	A	A	0
PI 94656	*T. dicoccon*	A	A	A	A	A	1/1+
PI 94657	*T. dicoccon*	B	B	A	A	A	0
PI 94664	*T. dicoccon*	A	A	A	A	A	1+
PI 94666	*T. dicoccon*	A	B	A	A	A	1+
PI 94674	*T. dicoccon*	A	B	A	A	A	1/1+
PI 94675	*T. dicoccon*	A	B	A	A	A	1
PI 154582	*T. dicoccon*	B	B	B	B	A	1/1+
PI 168679	*T. dicoccon*	A	B	A	A	A	1/1+
PI 193877	*T. dicoccon*	A	A	A	A	A	1-
PI 193879	*T. dicoccon*	A	A	A	A	A	1-
PI 193880	*T. dicoccon*	A	A	A	A	A	1-
PI 193882	*T. dicoccon*	A	A	A	A	A	1
PI 194042	*T. dicoccon*	A	A	A	A	A	1+
PI 194375	*T. dicoccon*	A	A	A	A	A	1+
PI 197259	*T. dicoccon*	A	A	A	A	A	1+/2
PI 197260	*T. dicoccon*	A	A	A	A	A	1/1+
PI 197481	*T. dicoccon*	A	A	A	A	A	1+/2
PI 197485	*T. dicoccon*	A	A	A	A	A	1-
PI 197489	*T. dicoccon*	A	A	A	A	A	2+/3
PI 197495	*T. dicoccon*	A	A	A	A	A	1
PI 217637	*T. dicoccon*	A	A	A	A	A	1+
PI 217639	*T. dicoccon*	A	B	A	A	A	1-/1
PI 221400	*T. dicoccon*	B	B	A	A	A	0
PI 225332	*T. dicoccon*	B	B	A	A	A	3

PI 244341	*T. dicoccon*	A	A	A	A	A	1+
PI 254147	*T. dicoccon*	A	A	A	A	A	1+
PI 254164	*T. dicoccon*	B	A	A	A	A	3
PI 254165	*T. dicoccon*	B	A	A	A	A	3
PI 254190	*T. dicoccon*	A	B	A	A	A	1-/1
PI 272531	*T. dicoccon*	A	B	A	A	A	1+
PI 273978	*T. dicoccon*	A	A	A	A	A	1+
PI 273980	*T. dicoccon*	A	A	A	A	A	4
PI 273981	*T. dicoccon*	A	A	A	A	A	1+
PI 275996	*T. dicoccon*	A	A	A	A	A	1+/2
PI 276013	*T. dicoccon*	No PCR product	B	A	B	A	3/3+
PI 298582	*T. dicoccon*	A	A	A	A	A	1/1+
PI 298586	*T. dicoccon*	A	A	A	A	A	1+
PI 324076	*T. dicoccon*	A	A	A	A	A	1+
PI 349043	*T. dicoccon*	A	B	A	A	A	1
PI 355477	*T. dicoccon*	A	A	A	A	A	1-
PI 355507	*T. dicoccon*	A	B	B	A	A	0
PI 362696	*T. dicoccon*	H	B	A	A	A	0
PI 377657	*T. dicoccon*	B	B	A	A	A	1
ISr8a-Ra	*T. aestivum*	A	No PCR product	A	A	A	3
ISr9a-Ra	*T. aestivum*	A	No PCR product	A	A	A	4
Avocet-S	*T. aestivum*	A	H	B	B	A	4
ISr5-Ra	*T. aestivum*	A	H	A	A	A	4
Jinan17	*T. aestivum*	A	H	A	B	A	4
Jimai229	*T. aestivum*	A	H	A	B	A	NT
Jimai24	*T. aestivum*	A	H	A	B	A	NT
Jimai44	*T. aestivum*	A	No PCR product	A	B	No PCR product	NT
Yannong19	*T. aestivum*	A	A	A	B	H	NT
Huaimai40	*T. aestivum*	A	A	A	A	A	4
Huaimai22	*T. aestivum*	A	H	A	A	A	NT
Huaimai44	*T. aestivum*	A	A	A	B	No PCR product	NT
Luyuan502	*T. aestivum*	A	A	A	A	A	4
Zhongmai895	*T. aestivum*	A	H	A	B	H	NT
Zhongmai578	*T. aestivum*	A	No PCR product	A	B	A	NT
Yumai70	*T. aestivum*	A	No PCR product	A	B	B	NT
Fielder	*T. aestivum*	H	B	B	B	A	4
Ningchun4	*T. aestivum*	A	H	A	A	A	4
Xiaoyan22	*T. aestivum*	A	A	A	A	A	NT
PI 660060	*T. aestivum*	H	B	B	B	A	NT
PI 600064	*T. aestivum*	H	A	A	B	A	NT
PI 679621	*T. aestivum*	A	No PCR product	A	A	A	NT
PI 648417	*T. aestivum*	A	A	A	A	A	NT
PI 442904	*T. aestivum*	A	A	A	B	A	NT
PI 603918	*T. aestivum*	A	A	A	B	A	NT

PI 189747	*T. aestivum*	H	B	H	B	A	NT
PI 660057	*T. aestivum*	A	A	A	B	A	NT
PI 182527	*T. aestivum*	B	B	H	B	A	NT
PI 600683	*T. aestivum*	A	A	A	B	A	NT
PI 660056	*T. aestivum*	A	A	B	B	A	NT
PI 679605	*T. aestivum*	A	A	A	B	No PCR product	NT
GSTR409	*T. aestivum*	A	A	B	B	A	NT
PI 675564	*T. aestivum*	H	B	B	B	A	NT
PI 672538	*T. aestivum*	A	A	A	B	A	NT
PI 277012	*T. aestivum*	A	A	A	A	A	NT
PI 596533	*T. aestivum*	A	A	A	A	A	NT
PI 679598	*T. aestivum*	A	A	B	B	A	NT
PI 679603	*T. aestivum*	H	B	H	B	A	NT
GSTR437	*T. aestivum*	A	A	B	B	A	NT
GSTR441	*T. aestivum*	A	A	A	A	A	NT
PI 638738	*T. aestivum*	A	A	A	A	A	NT
Taichang29	*T. aestivum*	A	A	A	B	A	NT
GSTR425	*T. aestivum*	A	A	B	B	A	NT
Xinong511	*T. aestivum*	A	A	A	B	A	NT
CItr15082	*T. aestivum*	A	A	No PCR product	B	A	NT
GSTR434	*T. aestivum*	A	A	B	B	A	NT
GSTR429	*T. aestivum*	A	A	B	B	A	NT
GSTR428	*T. aestivum*	A	A	B	B	A	NT
GSTR420	*T. aestivum*	A	A	B	B	A	NT
GSTR501	*T. aestivum*	A	No PCR product	A	A	A	NT
GSTR522	*T. aestivum*	A	A	A	B	A	NT
